# Brown Adipose Transplantation Improves Polycystic Ovary Syndrome-Involved Metabolome Remodeling

**DOI:** 10.3389/fendo.2021.747944

**Published:** 2021-11-29

**Authors:** Lihua Yao, Qin Wang, Runjie Zhang, Xingyun Wang, Yiwen Liu, Fangfang Di, Liwen Song, Siliang Xu

**Affiliations:** ^1^ Obstetrics and Gynecology Department, Tongren Hospital, Shanghai Jiao Tong University School of Medicine, Shanghai, China; ^2^ Hongqiao International Institute of Medicine, Tongren Hospital, Shanghai Jiao Tong University School of Medicine, Shanghai, China; ^3^ State Key Laboratory of Reproductive Medicine, Clinical Center of Reproductive Medicine, First Affiliated Hospital, Nanjing Medical University, Nanjing, China

**Keywords:** brown adipose tissue, metabolites, LC-MS, sphingosine, PCOS

## Abstract

Polycystic ovary syndrome (PCOS) is a complex reproductive, endocrine, and metabolic disorder in reproductive-age women. In order to explore the active metabolites of brown adipose tissue (BAT) transplantation in improving the reproductive and metabolic phenotypes in a PCOS rat model, the metabolites in the recipient’s BAT were explored using the liquid chromatography–mass spectrometry technique. In total, 9 upregulated and 13 downregulated metabolites were identified. They were roughly categorized into 12 distinct classes, mainly including glycerophosphoinositols, glycerophosphocholines, and sphingolipids. Ingenuity pathway analysis predicted that these differentially metabolites mainly target the PI3K/AKT, MAPK, and Wnt signaling pathways, which are closely associated with PCOS. Furthermore, one of these differential metabolites, sphingosine belonging to sphingolipids, was randomly selected for further experiments on a human granulosa-like tumor cell line (KGN). It significantly accelerated the apoptosis of KGN cells induced by dihydrotestosterone. Based on these findings, we speculated that metabolome changes are an important process for BAT transplantation in improving PCOS. It might be a novel therapeutic target for PCOS treatment.

## Introduction

Polycystic ovary syndrome (PCOS) is one of the most common endocrine diseases in reproductive-age women. Its prevalence varies from 9% to 18%, depending on different diagnostic criteria and ethnicity (1, 2). It is manifested as oligo-anovulation, hyperandrogenemia, and metabolic complications (3), accompanied with higher risks of type 2 diabetes mellitus and cardiovascular diseases (4). Lifestyle changes, including dietary modifications and exercise, are highly recommended as the first step of treatment. Medication alone cannot effectively treat the reproductive and metabolic symptoms of PCOS at the same time. For instance, metformin has been used to mainly ameliorate the metabolic manifestations of PCOS (5). Combined oral contraceptive pills have been the main treatment for PCOS patients for decades, with the disadvantages of a high risk of thrombosis and blood pressure anomalies (6, 7). To date, there is still a lack of effective treatment, whether a combination of lifestyle and medication interventions or alone, for PCOS ameliorating both reproductive and metabolic symptoms at the same time. Thus, it is necessary to explore novel therapies for PCOS.

Emerging evidence indicates that brown adipose tissue (BAT) transplantation plays a role in improving both the reproductive and metabolic phenotypes in PCOS animal models (8, 9). Interestingly, several studies suggest that BAT plays an endocrine role by secreting brown adipokines, such as growth and differentiation factor 15 and fibroblast growth factor 21 (9, 10). These brown adipokines could protect beta cell function, improve insulin sensitivity, and mediate the metabolic effects in obese mice (11, 12). Brown adipokines have been considered as candidate agents for therapeutic interventions in diverse metabolic diseases (10, 13, 14). In humans, classic BAT is abundant in newborns (15), which makes it difficult to study its effect on PCOS in adults. BAT is also abundant in small rodents. The identification of major brown adipokines and the characterization of their effects in animal models of PCOS are extremely important for the discovery of potential targets in PCOS.

Metabolomics has been redefined as the technology for discovering active factors of biological and pathological processes (16). Metabolomics can be harnessed to identify the metabolites that act as regulators of biological processes (17, 18), providing novel insights into the active role of metabolites in physiology and diseases. For example, phospholipids, sphingolipids, and methionine can act as regulators of insulin sensitivity and metabolism (19). Some metabolites, such as amino acids and sphingolipids, have also been regarded as biomarkers for the diagnosis of PCOS (20, 21). In addition, the metabolic disorder of arginine and proline may participate in the occurrence and development of PCOS (20), suggesting the roles of active metabolites in this disorder. However, the underlying mechanism of metabolites in PCOS remains unknown.

In this study, the protective effects of BAT transplantation on ovarian functions and metabolic disorders were investigated in a dehydroepiandrosterone (DHEA)-induced PCOS rat model. Non-targeted metabolomics were carried out to investigate the association between metabolites and BAT transplantation. The present study might provide a novel insight into the potential therapeutic effects and mechanistic actions of BAT transplantation in PCOS.

## Materials and Methods

### Establishment of DHEA-Induced PCOS Rat Model

All rat studies were approved by the Ethics Committee of Animal Experiments at Shanghai Tongren Hospital, Shanghai Jiao Tong University School of Medicine. The 21-day-old female Sprague–Dawley (SD) rats were purchased from China Three Gorges University Laboratory Animal Center, and all rats were allowed to adapt to the environment for 1 week. All rats were randomly divided into two groups: a control group (Ctrl) and a DHEA-induced PCOS model group (DHEA). Rats in the DHEA group were treated daily with a subcutaneous injection of DHEA (cat. no. SJ-HS0488, XXJL) for 21 days (6 mg/100 g bodyweight, dissolved in oil). The Ctrl group was injected with the same amount of oil. The successful PCOS model was determined by a significantly increased anti-Müllerian hormone (AMH) and luteinizing hormone/follicle-stimulating hormone (LH/FSH) ratio (8, 22, 23) and disordered estrous cycles. To verify the successful PCOS-like model, we randomly chose 5 control rats and 10 DHEA rats to test the levels of LH, FSH, and AMH. The estrous cycles were monitored for 10 days with a vaginal smear from day 11 to day 21 after DHEA or oil injection. In addition, two PCOS model rats and two control rats were randomly sacrificed to observe the ovarian morphology using hematoxylin and eosin (H&E) staining.

### Brown Adipose Tissue Transplantation and the Estrous Cycle Assessment

The PCOS model rats were randomly divided into two groups: a sham-operated (DHEA+Sham) group and a BAT transplantation (DHEA+BAT) group. Donor rats within 14 days after birth were operated on to take BAT (0.5 g of scapula), the peripheral white fat removed, and washed with sterile phosphate-buffered saline (PBS) at the same time. The recipient rats were intraperitoneally anesthetized. BAT was subcutaneously transplanted into the back of the recipient. For the DHEA+Sham group, the same procedure was used, except receiving donor tissues. These rats were kept for 3 weeks after operation. After 11 days of operation, the stages of the estrus cycle were determined by vaginal smear for consecutive 10 days. Finally, the rats were sacrificed to collect the BAT and ovarian tissues for further study (H&E staining, immunohistochemistry, and detection of metabolites).

### Sample Preparation

Approximately 50 mg of BAT was added into 0.5 ml of solvent (methanol/water = 8:2), containing 4 µg/ml 2-chloro-l-phenylalanine as an internal standard, and then ground, ultrasonicated at room temperature (25–28°C) for 10 min, and finally stored at −20°C for 30 min. After centrifugation at 13,000 rpm at 4°C for 10 min, 200 µl of the supernatant was taken for subsequent metabolomics analysis. Ten microliters of the supernatant from all samples was saved and mixed for quality control.

### Immunohistochemistry of Paraffin Section

The BAT and ovaries were fixed in 4% formaldehyde and embedded in paraffin; slices of 5 μm thickness were sectioned. After deparaffinization and rehydration, the sections were processed for blocking of endogenous peroxidase activity and antigen retrieval pretreatment, followed by blocking in 5% bovine serum albumin at room temperature for 20 min. The sections were then incubated overnight at 4°C with primary rabbit polyclonal uncoupling protein 1 (UCP1) antibodies (1:300, RRID: 72298; Cell Signaling Technology, Danvers, MA, USA). The second antibody was incubated for 30 min after washing three times with PBS. The signals were visualized with DAB incubation. Images were taken with the Digital Pathology Slide Scanner (KF-PRO-120, Ningbo Jiangfeng Medical Technology).

### Glucose Tolerance Test and Insulin Tolerance Test

The rats were fasted for 16 h (1700–0900 hours) with free access to drinking water and then injected intraperitoneally with d-glucose (2.0 g/kg body weight) for glucose tolerance test (GTT). Blood glucose levels were measured before the injection and at 15, 30, 60, 90, and 120 min after injection using an Accu-Chek glucose monitor (Roche Diagnostics Corp., Indianapolis, IN, USA). Female rats were fasted for 4 h (0900–1300 hours), with free access to drinking water, and injected intraperitoneally with insulin (1 U/kg body weight; Humulus; Eli Lilly, Indianapolis, IN, USA) for the insulin tolerance test (ITT). Blood glucose levels were measured before the injection and at 15, 30, 60, 90, and 120 min after insulin injection.

### Detection of Metabolic Profiling by LC-MS

Ultra-performance liquid chromatography (Ultimate 3000) combined with the Thermo Orbitrap Elite Mass Spectrometer was used for liquid chromatography–mass spectrometry (LC-MS) analysis. The flow rate was set to 0.4 ml/min with mobile phase A of 0.1% formic acid solution and mobile phase B of acetonitrile (0.1% formic acid). The column temperature was 25°C. Post time was set to 5 min to balance the system. MS uses the positive ion mode combined with the negative ion mode.

### Data Analysis

The Compound Discoverer software (Thermo Scientific, San Jose, CA, USA) was used to analyze the data. Post-editing was performed in EXCEL 2007 software. In order to obtain consistent differential variables, the resulting matrix was further optimized by removing all peaks with ion intensity = 0 in more than 80% of the samples. The data were normalized to the peak area of the corresponding internal standard, and the internal standard was used for reproducibility. Finally, the ion peaks generated by the internal standard were eliminated. Then, the edited data matrix was imported into Simca-P software (version 11.0). Before the multivariate statistical analysis, the data were mean centered and Pareto scaled. Principal component analysis (PCA) and partial least squares discriminant analysis (PLS-DA) were conducted to analyze the dissimilarity tendency among groups. Variable importance in projection (VIP) >1.0 and *p*-values <0.05 were selected as statistically significant according to the PLS-DA model. The Kyoto Encyclopedia of Genes and Genomes (KEGG) online database pathway enrichment analysis and ingenuity pathway analysis (IPA) were applied to understand the functions and interactions of the genes and metabolites in biological systems.

### Cell Culture

The steroidogenic human granulosa cell-like tumor cell line (KGN) was maintained in Dulbecco’s modified Eagle’s medium (DMEM)/F12 supplemented with 10% fetal bovine serum (FBS) (both from Gibco, Amarillo, TX, USA), 100 U/ml penicillin, and 100 μg/ml streptomycin in a humidified atmosphere at 37°C with 5% CO_2_. The growth medium was changed every 2–3 days.

### Apoptosis Analysis

Apoptosis was detected using an annexin V-FITC apoptosis detection kit (RRID: 556547; BD, Franklin Lakes, NJ, USA). KGN cells were seeded into six-well plates (3 × 10^5^ cells per well) and starved for 4 h in FBS-free medium. After stimulation with dihydrotestosterone (DHT) (Solarbio, Beijing, China) for 4 h, sphingosine was added (10 μm). The cells were detached using trypsin, washed with cold PBS twice, and then each well was incubated with 5 μl annexin V-FITC and 5 μl propidium iodide (PI) at room temperature for 15 min in the dark. Cells were detected by flow cytometry.

### Statistical Analysis

All the results were presented as the mean ± standard error of the mean (SEM). Univariate analysis of variance (ANOVA, with *post-hoc* Scheffe test) was applied to determine the significance of the observed differences among the Ctrl, DHEA+Sham, and DHEA+BAT groups using SPSS 26 for Windows (IBM, Armonk, NY, USA), whereas two groups were compared using unpaired Student’s *t*-test in the Ctrl and DHEA groups. GraphPad Prism 9.0 was used for other data analyses. A *p* < 0.05 was considered statistically significant.

## Results

### BAT Transplantation in PCOS Model Rat

**Figure 1:**
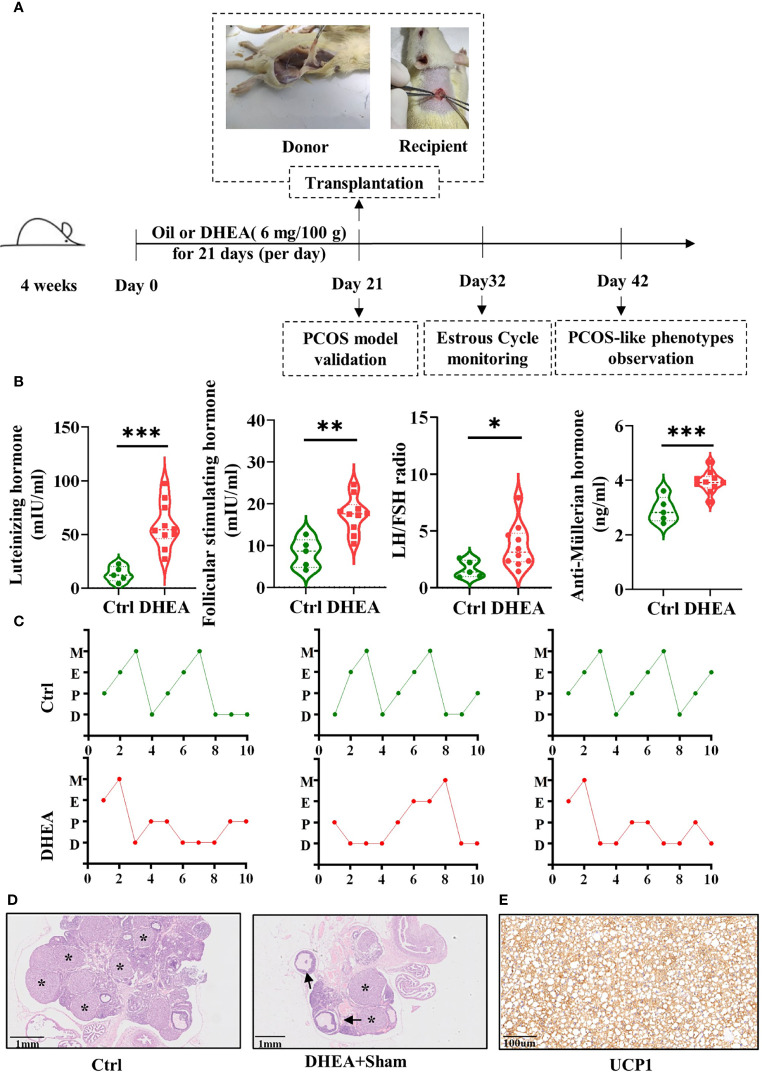
BAT transplantation in a PCOS rat model. **(A)** Scheme of the group assignments and timeline of the experiment process (*n* = 24). Female SD rats were treated with DHEA for 21 days to construct the PCOS model. BAT transplantation or sham operation was performed on day 21. Donor rats, within 14 days after birth, were operated on to take BAT and then BAT was transplanted to PCOS model rats. The estrous cycles were checked daily for the following 10 days. Reproductive and metabolism phenotype detection was done on day 42. **(B)** Serum concentrations of FSH, LH, and AMH, as well as the LH/FSH ratio (5 control rats and 10 DHEA rats). **(C)**. Disordered estrous cycles were observed in PCOS rats. **(D)** H&E staining of the ovarian tissues from the Ctrl and DHEA+Sham groups (*scale bar*, 1 mm). Ovarian histology revealed that cystic follicles (*arrow*) and a few corpora lutea (*asterisk*) appeared in the DHEA group compared with the Ctrl group. **(E)** UCP1 was identified by immunohistochemistry in the donor BAT (*scale bar*, 100 µm). *PCOS*, polycystic ovary syndrome; *BAT*, brown adipose tissue; *SD*, Sprague–Dawley; *DHEA*, dehydroepiandrosterone; *FSH*, follicle-stimulating hormone; *H&E*, hematoxylin and eosin; *LH*, luteinizing hormone; *AMH*, anti-Müllerian hormone; *UCP1*, uncoupling protein 1; D, diestrus; E, estrus; M, metestrus; P, proestrus. Data were analyzed using unpaired Student’s *t*-test. *p < 0.05, **p < 0.01, ***p < 0.001.

In order to determine the active adipokines of BAT related to PCOS, BAT transplantation in PCOS model rats was established. The experiment design is illustrated in **Figure 1A**. The PCOS model validation, estrous cycle monitoring, and PCOS-like phenotype observation were conducted in the scheme.

PCOS is always accompanied by altered plasma gonadotropin concentrations. The levels of LH, FSH, and AMH were tested to verify whether the PCOS model was a success. Compared with those in the Ctrl group, the concentrations of LH, FSH, and AMH and the LH/FSH ratio were significantly higher in the DHEA group (**Figure 1B**). The vaginal smear results of the rats showed disordered estrous cycles in the DHEA group (**Figure 1C**). Two PCOS model rats and two control rats were randomly sacrificed for H&E staining. H&E staining was performed to determine the alteration of ovarian pathology. In the DHEA group, multiple cystic follicles appeared with thinner granulosa cell layers, which were vacuolated and disordered in structure with corpus luteus (**Figure 1D**).

UCP1 is the critical regulator of thermogenesis, as a specific marker of BAT (24). The BAT of donor rats transplanted to rats in the DHEA+BAT group was verified by immunohistochemistry (**Figure 1E**). The results of UCP1 immunohistochemistry indicated that the transplanted tissues were BAT. The results mean the successful establishment of BAT transplantation in PCOS model rats.

### Effect of BAT Transplantation on the Follicular Development in the PCOS Model

**Figure 2:**
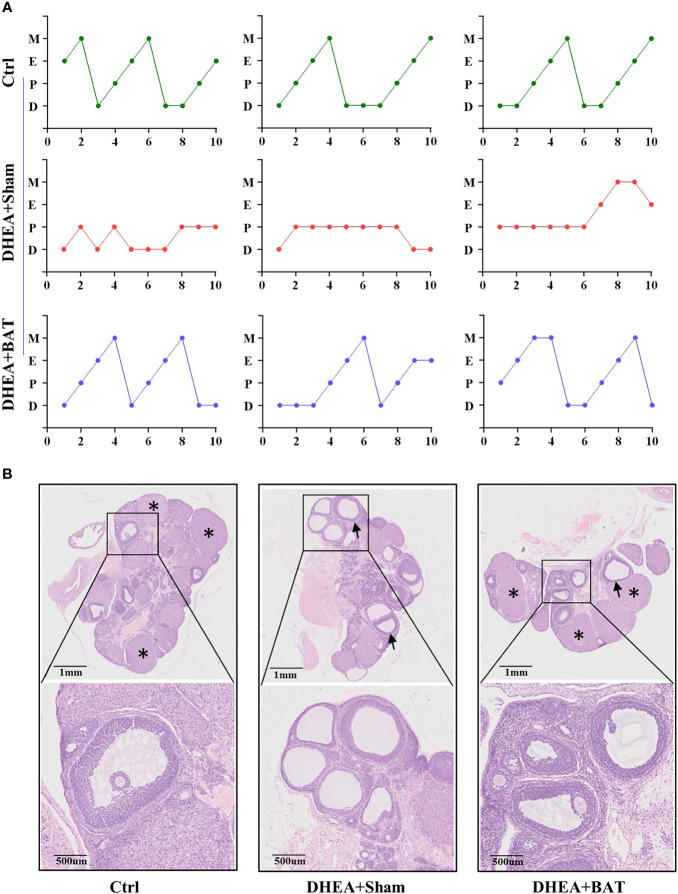
Brown adipose tissue (BAT) transplantation improved the reproductive phenotype of polycystic ovary syndrome (PCOS) model rats. **(A)** Disordered estrous cycles were observed in the DHEA+Sham group, while BAT transplantation rescued the abnormal estrous cycles. **(B)** Representative results of ovarian H&E staining of the control (Ctrl) group, DHEA+Sham group, and DHEA+BAT group. Ovarian histology revealed that cystic follicles (*arrow*) and a few corpora lutea (*asterisk*) appeared in the DHEA+Sham group compared with the Ctrl group, while BAT transplantation reversed the phenotype caused by dehydroepiandrosterone (DHEA). *H&E*, hematoxylin–eosin; *D*, diestrus; *E*, estrus; *M*, metestrus; *P*, proestrus; *UCP1*, uncoupling protein 1.

Irregular menstruation is one of the diagnostic criteria of PCOS. To explore whether BAT transplantation could recover the ovarian performance of PCOS rats, vaginal smear was performed to observe the effect of BAT transplantation on the estrous cycle in PCOS model rats (**Figure 2A**). In our study, we found that the Ctrl group had an ordered and complete estrous cycle, whereas a disordered estrous cycle manifested in the DHEA+Sham group. BAT transplantation rescued the phenotype of disordered estrous cycles in PCOS rats. Compared with the DHEA+Sham group, follicles of different stages and multiple luteal bodies reappeared with BAT transplantation (**Figure 2B**). Together, these results indicate that BAT transplantation can improve the ovarian performance of PCOS model rats.

### Effect of BAT Transplantation on Metabolic Characterization in the PCOS Model

**Figure 3:**
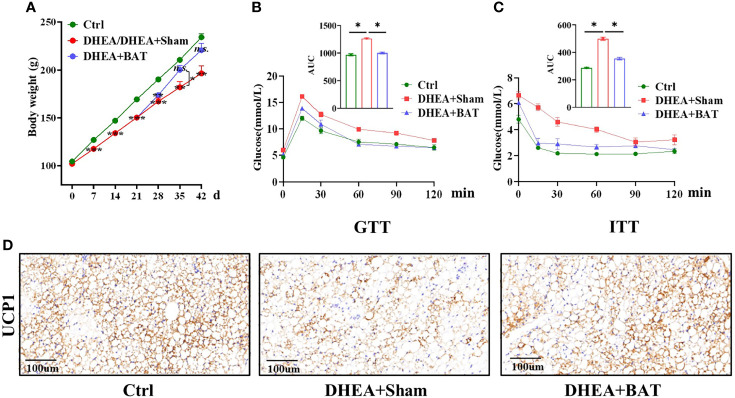
Brown adipose tissue (BAT) transplantation partially corrected the metabolic abnormality of polycystic ovary syndrome (PCOS) model rats. **(A)** Comparison of the mean weight among the control (Ctrl) group, the DHEA/DHEA+Sham group, and the DHEA+BAT group. **(B, C)** Results of the GTT **(B)** and ITT **(C)** showed that insulin resistance was rescued by BAT transplantation in PCOS model rats. **(D)** UCP1 expression was reduced in the DHEA+Sham group and was enhanced in the DHEA+BAT group. *GTT*, glucose tolerance test; *ITT*, insulin tolerance test; *UCP1*, uncoupling protein 1. Data were analyzed using one-way ANOVA with *post-hoc* Scheffe test. *p < 0.05, **p < 0.01, ***p < 0.001, *n.s.*, no significance.

Water and food were sufficiently provided for *ad libitum* intake. All rats were weighed every week, for a total of 6 weeks. The results showed that the mean weight of the DHEA group had significantly decreased compared to that of the Ctrl group on days 7, 14, and 21 before BAT transplantation (**Figure 3A**). The PCOS model rats were randomly divided into two groups on day 21: a DHEA+Sham group and a DHEA+BAT group. We compared the effects of BAT transplantation on the mean weight of PCOS model rats, and it was found that the mean weights of rats in the DHEA+BAT group increased compared with those in the DHEA+Sham group on days 28, 35, and 42. However, a significant difference was only observed on day 35 when comparing the DHEA+Sham group and the DHEA+BAT group.

PCOS is also caused by a metabolic disorder, which is characterized by impaired glucose tolerance and insulin tolerance. The GTT was performed on day 39 and the ITT performed on day 42 to observe the metabolic changes. The GTT results showed a delayed glucose clearance in the DHEA+Sham group, and BAT transplantation partially reversed it (**Figure 3B**). For the ITT, the DHEA+Sham group had higher glucose levels (**Figure 3C**). In addition, the UCP1 levels decreased in the DHEA+Sham group and were recovered in the DHEA+BAT group (**Figure 3D**). These results indicate that BAT transplantation ameliorates insulin resistance and corrects the metabolic abnormality in PCOS model rats.

### The Differential Changes in Metabolomics Resulting From BAT Transplantation

**Figure 4:**
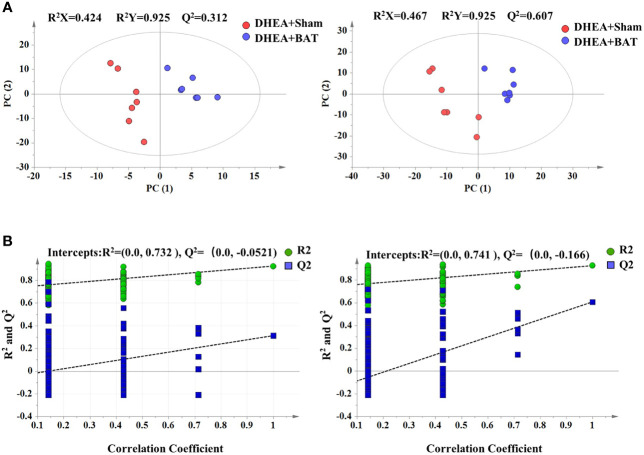
Score plot of the PLS-DA model. **(A)** Score plot of the PLS-DA model in the positive and the negative mode. **(B)** Permutation test of the PLS-DA model in the positive and the negative mode. *Green dots* indicate *R* ^2^ and *blue dots* indicate *Q* ^2^. *PLS-DA*, partial least squares discriminant analysis.

BAT transplantation could activate the recipient’s BAT function; thus, there are metabolic differences between the DHEA+Sham and DHEA+BAT groups. In order to identify the active metabolites of the recipient’s BAT, a series of multivariate variable pattern recognition analyses were carried out using LC-MS. Firstly, PCA was established to determine the separation tendency between the DHEA+Sham and DHEA+BAT groups (**Supplementary Figure S1**). A two-component PCA model was obtained with the following parameters: *R* ^2^ *X* = 0.506, *Q* ^2^ = 0.284 (positive mode) and *R* ^2^ *X* = 0.517, *Q* ^2^ = 0.364 (negative mode). To further specify the metabolic variations associated with PCOS, a supervised PLS-DA model was established with two predictive components (positive mode: *R* ^2^ *X* = 0.424, *R* ^2^ *Y* = 0.925, *Q* ^2^ = 0.312; negative mode: *R* ^2^ *X* = 0.467, *R* ^2^ *Y* = 0.925, *Q* ^2^ = 0.607). As shown in **Figure 4A**, a clear separation was obtained in the scores plot, with all the DHEA+Sham in the left half and DHEA+BAT in the right half. To further validate the established model, a 200-time permutation test was performed for the PLS-DA model. The *Y*-axis intercept for *Q* ^2^ was below 0 [*Q*_2_ intercept (0, −0.0521) (positive mode) and (0, −0.166) (negative mode)] (**Figure 4B**). These results validate the current supervised model. The PLS-DA results demonstrated significant metabolic differences between the DHEA+Sham and DHEA+BAT groups. Moreover, significant biochemical changes were induced by BAT transplantation.

### Enrichment Analysis on Differential Metabolites

**Figure 5:**
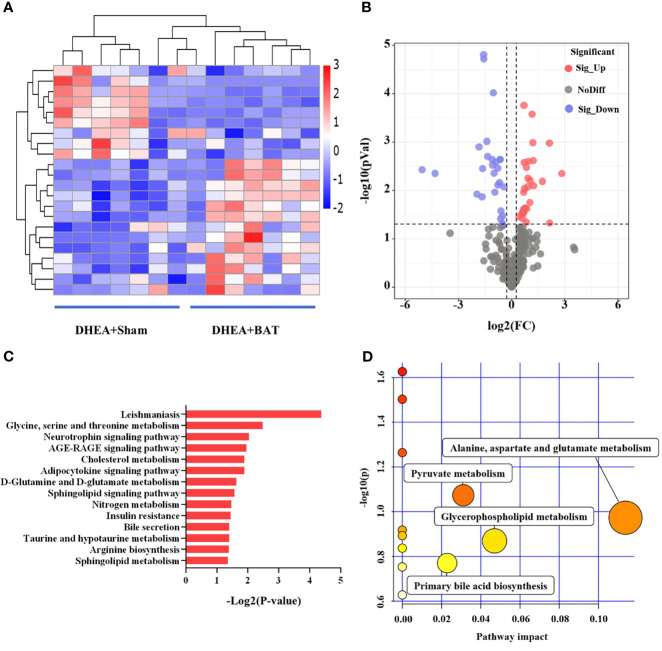
Differential analysis of the metabolites in brown adipose tissue (BAT) from the DHEA+Sham and DHEA+BAT groups. **(A)** Heatmap of the 22 metabolites that were differentially expressed between the DHEA+Sham (*n* = 7) and DHEA+BAT (*n* = 7) groups. *Blue* to *red* equates to an increase in metabolite expression. **(B)** Volcano plot of all metabolites expressed in the DHEA+Sham and DHEA+BAT groups. **(C)** KEGG pathway analysis of the differentially expressed metabolites. **(D)** All matched pathways are displayed as *circles* analyzed with MetaboAnalyst 3.0. Potential target pathways were selected either by impact values from pathway topology analysis or by negative log *p*-values from pathway enrichment analysis. The *size of the bubble* represents the number of metabolites enriched. *KEGG*, Kyoto Encyclopedia of Genes and Genomes.

A total of 321 metabolites were identified in this study. Further metabolomics analysis identified a total of 22 differential metabolites (**Table 1** **)**, including 9 upregulated and 13 downregulated metabolites, when comparing the DHEA+BAT group with the DHEA+Sham group. The differential metabolites with VIP > 1 and *p* < 0.05 were clustered and shown as a heatmap between the DHEA+Sham and DHEA+BAT groups (**Figure 5A**). The results were visualized in a volcano plot of all metabolites, as shown in **Figure 5B**. KEGG pathway enrichment analysis of the differential metabolites showed their connection with the adipocytokine signaling pathway, insulin resistance, etc. (**Figure 5C**). Important analysis of the metabolic pathways in the bubble diagram showed that the differential metabolites were associated with amino acid metabolism, glycerophospholipid metabolism, and pyruvate metabolism (**Figure 5D**).

**Table 1:** Dysregulated metabolites between the DHEA+Sham and DHEA+BAT groups.

Class	Name	VIP	*p*-value	Fold change
Sphingolipids	*N*-palmitoyl-d-erythro-sphingosine	1.32187	0.048360335	0.701984889
Dehydes	Pyruvaldehyde	1.4637	0.002293146	0.641374704
Amino acids	dl-β-Leucine	1.03371	0.011904801	0.257662834
	l-Glutamine	1.38227	0.006913317	0.627002081
Cholic acid	Taurocholic acid	1.03748	0.004480277	7.0752304
Fatty acyls	1-Linoleoyl glycerol	1.13363	0.008396951	1.889026271
Fatty esters	Icosadienoic acid	1.23869	0.00649719	3.345072531
Glycerophosphocholines	Lyso-PAF C-16	1.3197	0.03773069	1.525340715
	PC(P-15:0/0:0)	1.09853	0.024659805	1.587064544
Glycerophosphoethanolamines	LysoPE(0:0/22:4(7Z,10Z,13Z,16Z))	1.474	0.005592236	1.897683421
Glycerophosphoinositols	PI(18:0/22:5(4Z,7Z,10Z,13Z,16Z))	1.20277	0.003526964	0.324269691
	PI(18:0/22:6(4Z,7Z,10Z,13Z,16Z,19Z))	1.45847	0.013576691	0.315352317
	PS(18:0/22:5(7Z,10Z,13Z,16Z,19Z))	1.16498	0.003497396	0.585825926
Glycerophosphates	beta-Glycerophosphoricacid	1.16314	0.004377947	0.502442175
Peptides	Asp Ile Lys Arg	1.03604	0.030470895	1.509817996
	Cys Gln Trp Trp	1.24675	0.002413147	2.344734335
	Leu Asp	1.22868	0.047455033	4.396481329
	Phe Phe Arg Arg	1.29797	0.000174435	1.620741001
	Tyr Lys Val Glu Ile	1.26152	0.0108703	1.614132279
Phenylsulfates	4-Ethylphenylsulfate	1.66923	0.002512745	1.917318971
TCA	d-(+)-Malic acid	1.14599	0.030182727	1.516545227
Unclassified	Glc-GP(18:0/20:4(5Z,8Z,11Z,14Z))	1.33843	0.001968024	0.390099725

DHEA, dehydroepiandrosterone; BAT, brown adipose tissue; VIP, variable importance in projection; TCA, trichloroacetic acid.

### Downstream Analysis of the Differential Metabolites by Ingenuity Pathway Analysis

**Figure 6:**
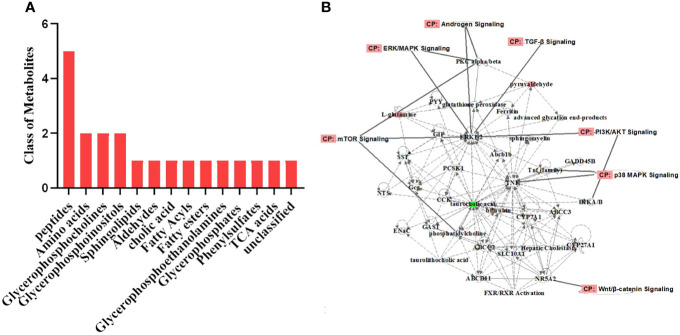
Classification of the differential metabolites and IPA. **(A)** Classification of the differential metabolites was roughly categorized into 12 distinct classes. **(B)** IPA of the metabolites related to biological network, pathways, and functions. The differential metabolites were closely associated with the PI3K/AKT, MAPK, and Wnt signaling pathways. *IPA*, ingenuity pathway analysis.

The differential metabolites were roughly categorized into 12 distinct classes, mainly including glycerophosphoinositols, glycerophosphocholines, and sphingolipids (**Figure 6A**). IPA of the differential metabolites revealed several related signaling pathways, such as the PI3K/AKT, MAPK, and Wnt signaling pathways, which have been proven to be closely associated with PCOS (25–27) (**Figure 6B**). These altered metabolites might explain the positive effect of BAT transplantation on PCOS model rats.

### Identification of Nine Differential Metabolites After BAT Transplantation

**Figure 7:**
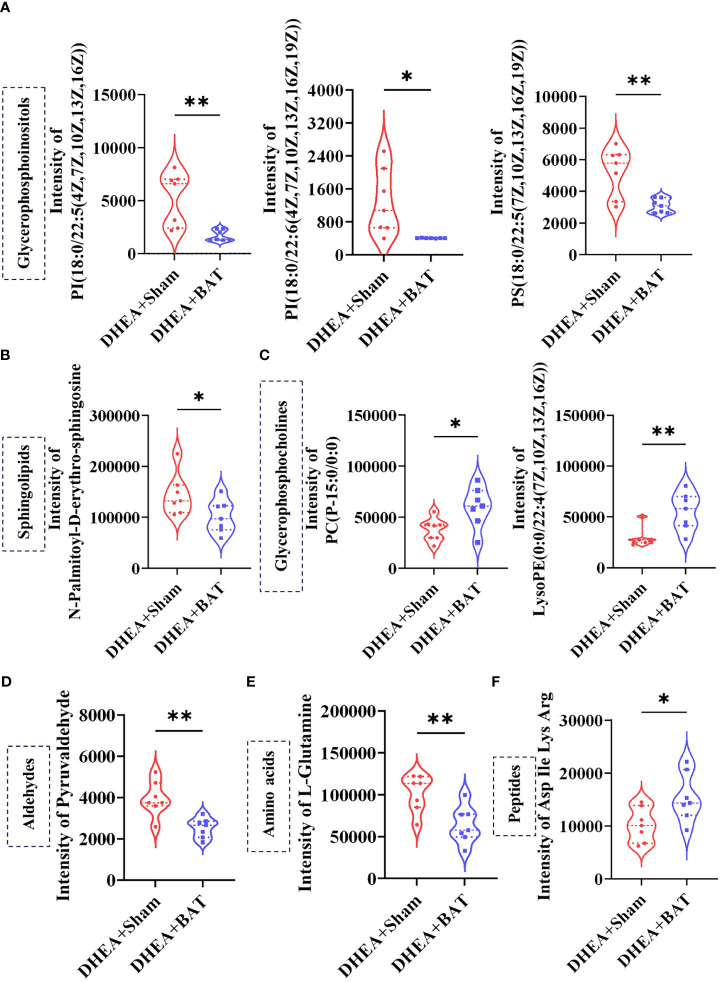
Selected differentially expressed metabolites. **(A–C)** The glycerophosphoinositols and sphingolipids were decreased, while glycerophosphocholines were increased in the DHEA+BAT group. **(D, E)** Aldehydes and amino acids were reduced in the DHEA+BAT group compared with that in the DHEA+Sham group. **(F)** Peptides were increased in the DHEA+BAT group. Data were analyzed using unpaired Student’s *t*-test. *p < 0.05, **p < 0.01.

The relative differences among the metabolite classes revealed significant changes in the abundance of metabolites. Glycerophosphoinositols, sphingolipids, aldehydes, and amino acids were decreased (**Figures 7A, B, D, E**), while glycerophosphocholines and peptides were increased in the DHEA+BAT group (**Figures 7C, F**).

### The Effect of Sphingosine on the Apoptosis of KGN Cells

**Figure 8:**
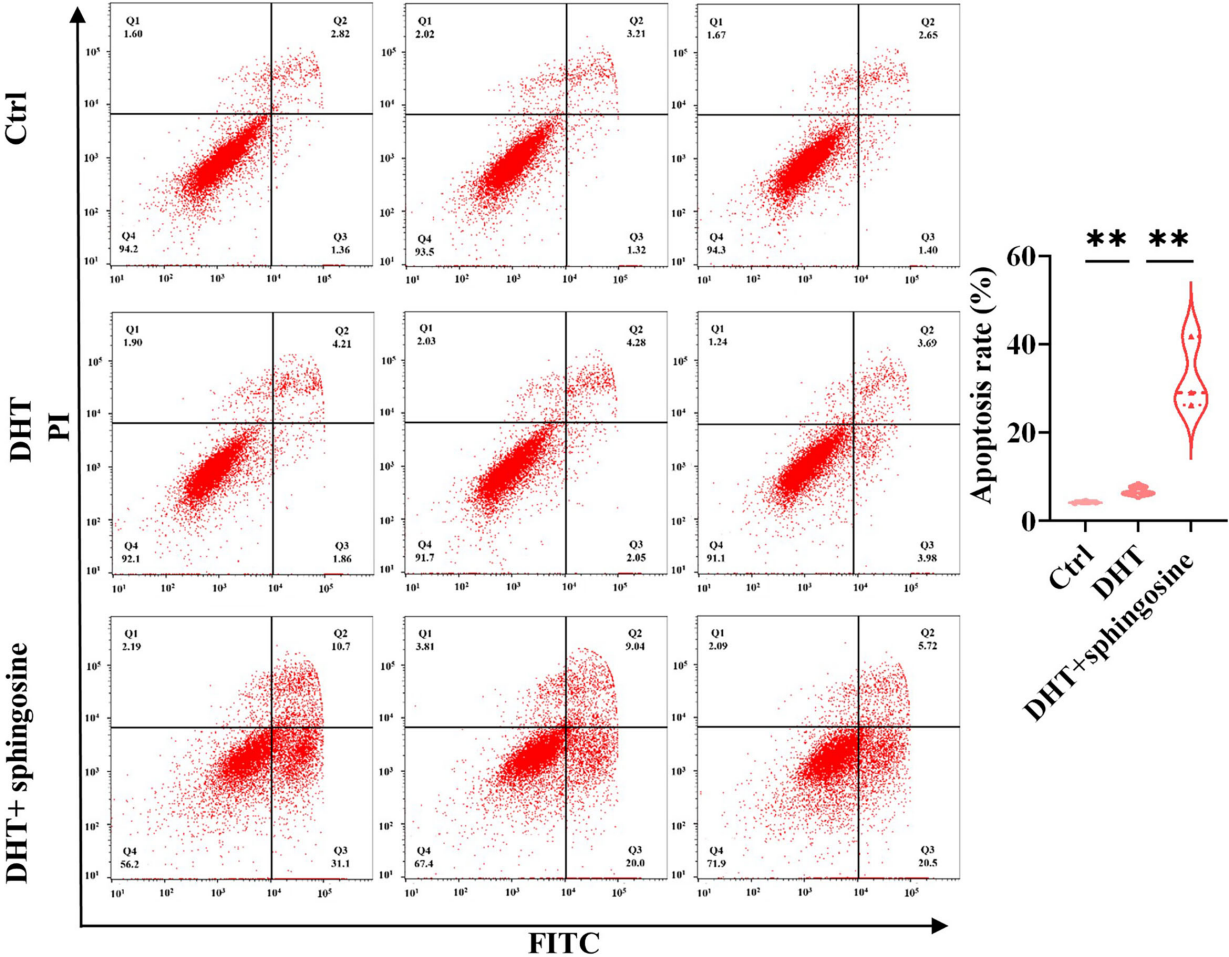
Analysis of the cell apoptosis of KGN cells transfected with sphingosine. The early and late apoptosis rates of KGN cells were compared using PI and FITC. Sphingosine increased the apoptosis of DHT-treated KGN cells. The results are triplicates. *DHT*, dihydrotestosterone; *PI*, propidium iodide; *FITC*, fluorescein isothiocyanate. Data were analyzed with unpaired Student’s *t*-test. **p < 0.01.

To further explore the biological roles of metabolites, we randomly chose the differential metabolite sphingosine. KGN cells were pretreated with DHT to mimic the pathophysiological status of PCOS. DHT has been confirmed to be capable of inducing rodent models to exhibit similar reproductive and metabolic features to PCOS patients (28) and has been used in KGN cells at a dosage of 25 nM to imitate the physiological characteristics of PCOS (29). Our results showed that sphingosine significantly increased the apoptosis of DHT-treated KGN cells (**Figure 8**).

## Discussion

In this study, we verified that BAT transplantation successfully rescued the metabolic symptoms of PCOS model rats and ameliorated the DHEA-induced ovarian dysfunction and abnormal metabolism. Our study indicated that BAT transplantation might have a protective effect on the metabolome remodeling of the recipient’s BAT in PCOS rats. In addition, the differential metabolites were identified by LC-MS, mainly including glycerophosphoinositols, glycerophosphocholines, sphingolipids, and amino acids. The IPA demonstrated that the metabolites were associated with the PI3K/AKT, MAPK, and Wnt signaling pathways, which play important roles in the pathogenesis of PCOS. Furthermore, we found that one of the metabolites, sphingosine, could enhance the apoptosis of DHT-induced KGN cells, suggesting that altered metabolites in the recipient’s BAT could affect the function of granulosa cells. Our study provides new insights into the therapeutic potential bioactive metabolites in PCOS treatment.

In our study, multiple cystic follicles with thinner granulosa cell layer were observed in the DHEA group, which may be related to the increased apoptosis of granulosa cells (30). However, the level of AMH was higher in the DHEA group compared with that in the Ctrl group. As a matter of fact, AMH is known to be mainly produced by the granulosa cells of pre-antral and antral follicles. Since increased numbers of small follicles in the pre-antral and antral stages are observed in PCOS patients, the level of AMH in PCOS patients is higher than that in women with normal ovaries. Furthermore, individual granulosa cells produce more AMH in PCOS by calculating the ratio of AMH to antral follicle counts (31, 32). Consistently, in this study, multiple cystic follicles were observed in the DHEA group, which may produce more AMH by individual granulosa cells similar to that in PCOS patients. In addition, it is likely that the apoptotic granulosa cells belong to bigger follicles and that the smaller follicles are preserved, which further result in more AMH production in the DHEA group.

Although an impaired glucose regulation was observed in the DHEA group, the body weight was also decreased unexpectedly in our study. According to existing research, some studies indicated that DHEA can increase the body weight, while other studies showed that the DHEA group could have a tendency to lose weight as well (33, 34). We speculated that it may be related to the following aspects: 1) a low dose of DHEA could prevent the development of obesity (35–37); 2) DHEA can reduce the food intake of rodents (38); 3) experimental procedures such as injection therapy or vaginal smears invisibly increase the amount of exercise. In addition, PCOS patients include the lean and obese types, both of which are accompanied by impaired glucose tolerance or insulin resistance. Although our PCOS model did not show obesity, we observed impaired glucose regulation, estrous cycle disturbance, and multiple cystic follicles, which met the modeling standards of PCOS. However, this may be a limitation of our study, and the underlying mechanism still needs further exploration.

Several studies have revealed the reduced activity of BAT in women with PCOS (39). BAT transplantation, a promising therapeutic strategy, has been used to prolong the ovarian life span in animal models (40). Besides, BAT transplantation was applied for the treatment of metabolic diseases such as obesity and cardiovascular disease (41, 42). Our results showed that BAT transplantation could ameliorate the reproductive and metabolic phenotype by the improving the metabolic function of the recipient’s BAT in PCOS rats. BAT can act as an endocrine organ to affect whole-body metabolism and release adipokines that improve glucose metabolism (43). These indicate that BAT transplantation may recover the ovarian function and metabolic disorder by secreting bioactive adipokines in PCOS rats.

Metabolomics is a large-scale study that examines the relationship between specific metabolites and diseases. Emerging evidence indicates that metabolites play important roles and may act as therapeutic tools in diverse diseases (16). We conducted the metabolomics of the recipient’s BAT with LC-MS and identified 22 differential metabolites. They were mainly classified as the glycerophospholipid family, the sphingolipid family, the amino acid and peptide family, and the aldehyde family. The glycerophospholipids, such as glycerophosphoinositols and glycerophosphocholines, are the lipid components of cell membranes that participate in a variety of indispensable metabolic and intracellular signaling processes (44). Our results showed that glycerophosphoinositols were downregulated in the DHEA+BAT group. In addition, phosphocholines (PCs), one of the glycerophosphocholines, were decreased in the DHEA+Sham group compared with that in the DHEA+BAT group. Combined with the published results of reduced PC levels in PCOS patients (45, 46), it can be speculated that PC played an important role in the protective effects of BAT transplantation for PCOS. Sphingolipids are also part of the membrane lipids that are involved in many biological processes, such as cell proliferation, apoptosis, and differentiation (47). Sphingosine, one type of sphingolipid that decreased in the DHEA+BAT group, has anti-proliferative and pro-apoptotic effects (48, 49), and it may have exerted its function in the process of BAT transplantation.

Using KEGG pathway analysis, we found that the differential metabolites were mainly enriched in amino acid metabolism, adipocytokine signaling pathway, insulin resistance pathway, and sphingolipid signaling pathway. Moreover, the metabolic pathways showed that amino acid metabolism, glycerophospholipid metabolism, and pyruvate metabolism were associated with BAT transplantation in PCOS rats. In addition, we found various pathways associated with BAT transplantation, mainly enriched in amino acid metabolism. Some studies demonstrated that amino acid disorder is associated with obesity, insulin resistance, and type 2 diabetes mellitus (50, 51). Recent studies have also found that women with PCOS suffer from amino acid metabolism disorders (52, 53). These results indicate that amino acid metabolism may play an important role in the process of BAT transplantation in PCOS rats. In addition, IPA revealed that the differential metabolites were closely related with the PI3K/AKT, MAPK, and Wnt signaling pathways. The PI3K/AKT signaling pathway is involved in several critical regulators of granulosa cell proliferation and differentiation (54), the dysregulation of which may contribute to impaired follicular development. Furthermore, abnormal PI3K/AKT signaling pathway is also closely related to insulin resistance, abnormal follicle development, and metabolic disorders in PCOS (25). In PCOS patients, aberrant MAPK signaling contributes to the dysregulation of granulosa cell proliferation, metabolic disorder, and overproduction of ovarian androgen (55, 56). These suggest that the differential metabolites may play roles in the process of BAT transplantation in PCOS rats. Our study provides preliminary evidence that metabolites link the association between BAT transplantation and PCOS.

One of the pathological features of PCOS is abnormal follicular development. The augmented apoptosis of granulosa cells may have a key role in the pathogenesis of PCOS (57, 58). Sphingosine could inhibit cell growth and induce cell apoptosis (59). Our results demonstrated that sphingosine was increased in the DHEA+Sham group and that BAT transplantation could reduce its level. The KGN cell experiment showed that sphingosine might enhance the apoptosis of KGN cells, indicating that altered metabolites play a role in the process of BAT transplantation in the treatment of PCOS.

In conclusion, we revealed the positive role of the recipient’s BAT in ameliorating PCOS. The altered metabolites were closely associated with the PI3K/AKT and MAPK signaling pathways. In addition, we discovered that sphingosine enhanced the apoptosis of granulosa cells in PCOS. All these results indicate that the beneficial effects of BAT transplantation are partly mediated by the bioactive metabolites. Herein, our study provides a new light on the potential therapeutic strategy for PCOS.

## Data Availability Statement

The datasets presented in this study can be found in online repositories. The names of the repository/repositories and accession number(s) can be found in the article/**Supplementary Material**.

## Ethics Statement

The animal study was reviewed and approved by the Ethics Committee of Animal Experiments at Shanghai Tongren Hospital, Shanghai Jiao Tong University School of Medicine.

## Author Contributions

LY and LS designed the research. LY, QW, RZ, and YL performed the experiments. LY, FD, XW, and LS analyzed the data. LY, XW, SX, and LS wrote the article. All authors contributed to manuscript revision, read, and approved the submitted version.

## Funding

This study was supported by the Scientific Research Project of Shanghai Municipal Health Commission (grant no. 20194Y007), National Natural Science Foundation of China (grant nos. 82001508 and 81903006), the Natural Science Foundation of Jiangsu Province (grant no. BK20181089), and the Shanghai Changning District Science and Technology Commission Project (grant no. CNKW2018Y06).

## Conflict of Interest

The authors declare that the research was conducted in the absence of any commercial or financial relationships that could be construed as a potential conflict of interest.

## Publisher’s Note

All claims expressed in this article are solely those of the authors and do not necessarily represent those of their affiliated organizations, or those of the publisher, the editors and the reviewers. Any product that may be evaluated in this article, or claim that may be made by its manufacturer, is not guaranteed or endorsed by the publisher.

## Supplementary Material

Supplementary Figure 1 PCA scores plot showed a distinct metabolome profile of DHEA+ Sham group compared with DHEA+BAT group in the positive mode and the negative mode. X-axis and Y-axis represented the first and second principal components, respectively. QC, quality control; PCA, principal component analysis.

Supplementary Figure 2 H&E staining of the ovarian tissues. **(A)**. Another H&E staining of the ovarian tissues from Ctrl and DHEA+Sham groups (scale bar=1mm). Ovarian histology revealed that cystic follicles (arrow) and few corpora lutea (asterisk) appeared in the DHEA group compared with Ctrl group. **(B)**. Another representative results of ovarian H&E staining of the Ctrl group, DHEA+Sham group, and DHEA+BAT group. Ovarian histology revealed that cystic follicles (arrow) and few corpora lutea (asterisk) appeared in the DHEA+Sham group compared with Ctrl group, while BAT transplantation reversed the phenotype caused by DHEA.
