# Supplementary figures and images for: Brown Adipose Transplantation Improves Polycystic Ovary Syndrome-Involved Metabolome Remodeling
Source: Front Endocrinol (Lausanne). 2021 Nov 29;12:747944. doi: 10.3389/fendo.2021.747944 (PMC8667175; doi:10.3389/fendo.2021.747944)

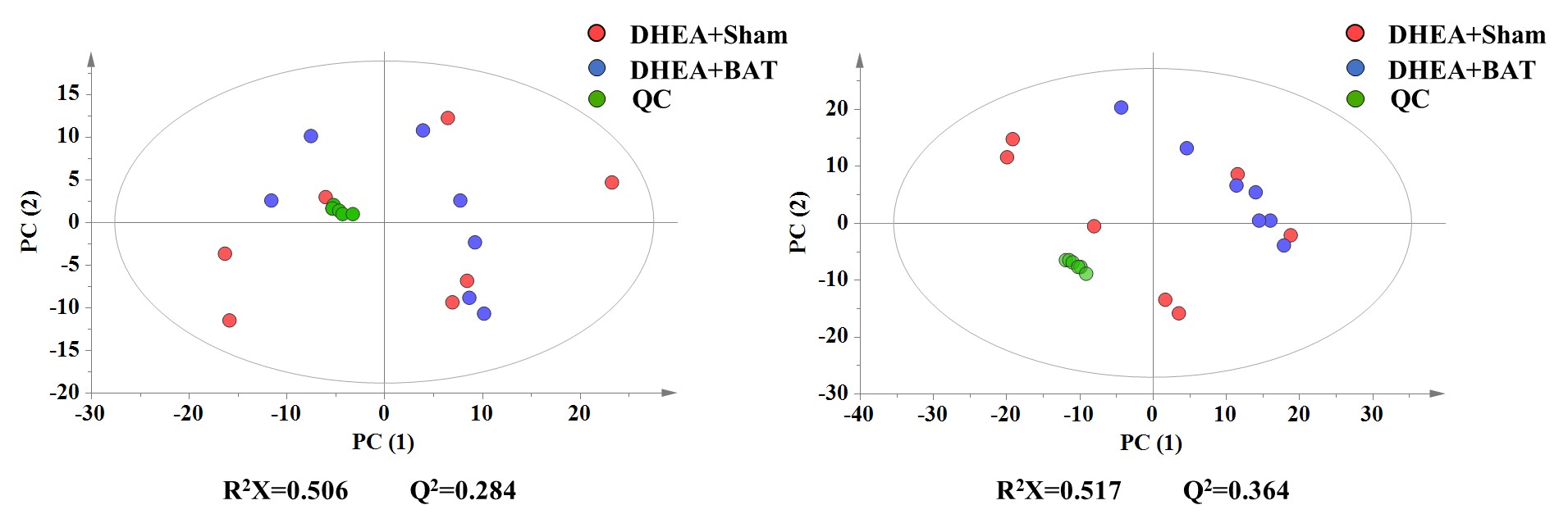

Supplement: Supplementary Figure 1 — PCA scores plot showed a distinct metabolome profile of DHEA+ Sham group compared with DHEA+BAT group in the positive mode and the negative mode. X-axis and Y-axis represented the first and second principal components, respectively. QC, quality control; PCA, principal component analysis. [file Image_1.jpeg]

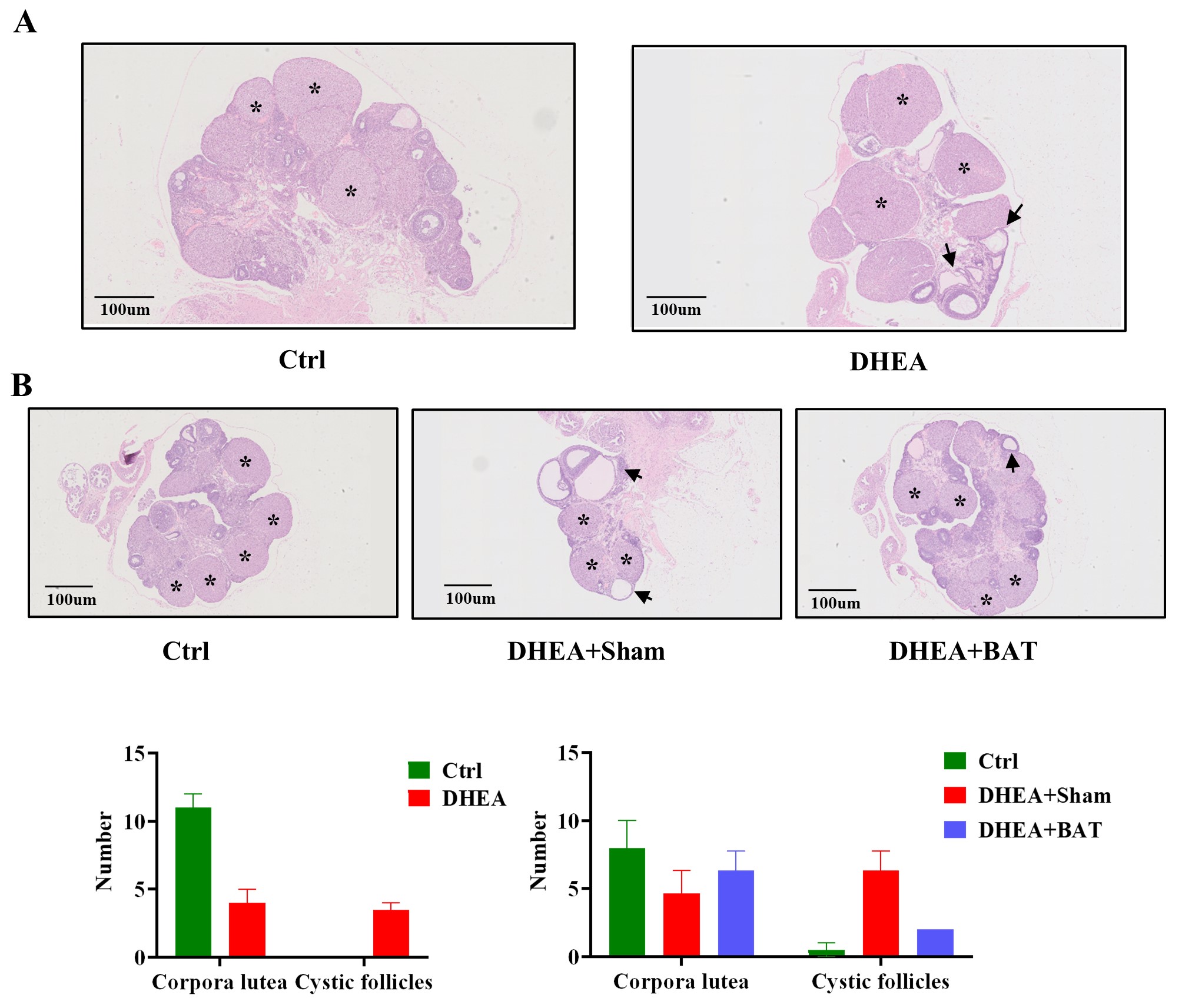

Supplement: Supplementary Figure 2 — H&E staining of the ovarian tissues. (A). Another H&E staining of the ovarian tissues from Ctrl and DHEA+Sham groups (scale bar=1mm). Ovarian histology revealed that cystic follicles (arrow) and few corpora lutea (asterisk) appeared in the DHEA group compared with Ctrl group. (B). Another representative results of ovarian H&E staining of the Ctrl group, DHEA+Sham group, and DHEA+BAT group. Ovarian histology revealed that cystic follicles (arrow) and few corpora lutea (asterisk) appeared in the DHEA+Sham group compared with Ctrl group, while BAT transplantation reversed the phenotype caused by DHEA. [file Image_2.jpeg]
